# Longitudinal associations of depressive and burnout symptoms with eating behavior and physical activity in occupational health clients with obesity

**DOI:** 10.1080/21642850.2026.2700076

**Published:** 2026-08-05

**Authors:** Siniriikka A. Männistö, Joona Muotka, Laura-Unnukka Suojanen, Raimo Lappalainen, Kirsi H. Pietiläinen, Riitta Korpela

**Affiliations:** a Obesity Research Unit, Research Program for Clinical and Molecular Metabolism, Faculty of Medicine, University of Helsinki, Helsinki, Finland; b Healthy Weight Hub, Abdominal Centre, Helsinki University Hospital and University of Helsinki, Helsinki, Finland; c Department of Psychology, University of Jyväskylä, Jyväskylä, Finland; d Department of Pharmacology, Faculty of Medicine, University of Helsinki, Helsinki, Finland

**Keywords:** Depression, occupational burnout, emotional eating, eating competence, physical activity

## Abstract

**Introduction:**

Depression and occupational burnout are prevalent mental health challenges in working-age populations and are linked to adverse health behaviors, with these concerns particularly relevant among individuals with obesity. Although prior research suggests associations with eating behaviors and physical activity, most studies rely on cross-sectional designs, leaving the temporal and potentially bidirectional relationships between mental health and health behaviors unclear.

**Methods:**

This longitudinal study examined bidirectional associations between depressive and burnout symptoms, eating behavior, and physical activity among occupational health clients with obesity (*N* = 110; BMI 30–40 kg/m^2^) participating in a 12-month behavioral intervention. At baseline, the mean age was 50.8 years and mean BMI 34.2 kg/m^2^; 17% were male and most were white-collar workers. Assessments at baseline, 6, and 12 months included depression (BDI-21), burnout (BBI-15), eating competence (ecSI 2.0), dietary behaviors (TFEQ-R18), and physical activity (BHPAQ). A random-intercept cross-lagged panel model (RI-CLPM) was used to examine within-person longitudinal associations.

**Results:**

Higher depressive symptoms predicted subsequent decreases in eating competence and increases in emotional eating, although the depression–emotional eating association was derived from a traditional CLPM rather than an RI-CLPM model due to convergence issues. Elevated burnout symptoms predicted subsequent increases in emotional eating and decreases in physical activity. In addition, emotional eating predicted later burnout symptoms. No longitudinal associations were observed between depression and physical activity, or between depression or burnout and uncontrolled eating.

**Discussion:**

Elevated depressive and burnout symptoms may predispose individuals to less healthy behaviors, potentially limiting the effectiveness of health interventions. These findings highlight the interplay between mental health, eating, and physical activity, suggesting that addressing psychological distress in behavior change programs may improve outcomes for individuals with elevated depression and burnout.

**Clinical trials:**

gov NCT04785586; RR2-10.1016/j.conctc.2020.100638.

## Introduction

Depression and occupational burnout are prevalent and interrelated forms of psychological distress in working-age populations (Tóth‐Király et al., 2021). While depression reflects broader emotional dysfunction, burnout is a work-related phenomenon characterised by exhaustion, cynicism, and reduced professional efficacy (Maslach et al., 2001; Tolentino & Schmidt, 2018; WHO, 2024).

Growing evidence links depression and burnout to disturbances in health behaviours (e.g. Firth et al., 2020; Hanson et al., 2016; Mincarone et al., 2024; Penttinen et al., 2021). More specifically, research has shown that depression is associated with maladaptive eating patterns, particularly emotional and uncontrolled eating (Konttinen et al., 2019; Paans et al., 2018), while evidence on burnout suggests similar but less studied associations (Nevanperä et al., 2012). Physical activity, in turn, appears to be inversely and bidirectionally associated with depression and is also linked to burnout symptoms (Schuch et al., 2018; Shoman et al., 2021; Zhang et al., 2021).

Individuals with obesity represent a particularly important population for examining the interplay between psychological distress and health behaviours. Obesity is associated with higher rates of depressive symptoms, emotional eating, reduced physical activity, and difficulties maintaining long-term behavioural change (Avila et al., 2015; Benbaibeche et al., 2023; Nordmo et al., 2020; Silveira et al., 2022). Emerging evidence also suggests that occupational burnout may contribute to obesity development and weight gain through stress-related behavioural pathways (Kremers et al., 2025; Tomiyama, 2019). These relationships are especially relevant in occupational health settings, where obesity, mental well-being, work ability, and productivity are closely intertwined.

Although depression and burnout share several features, they represent partly distinct forms of psychological distress (Tóth‐Király et al., 2021). Understanding whether these conditions differentially influence eating behaviour and physical activity may help occupational health services better identify individuals at risk of unsuccessful behaviour change and provide more targeted support.

Most previous studies have relied on cross-sectional or between-person analyses. Consequently, little is known about whether changes in depressive or burnout symptoms within an individual precede subsequent changes in eating behaviour or physical activity. Addressing this question may provide important insights into mechanisms that influence the success of behavioural obesity interventions.

Therefore, the study aimed to explore the longitudinal associations between depression and burnout and their relationships with eating behaviour and physical activity during a 12-month behavioural intervention among occupational health patients with obesity. Specifically, we examined whether changes in depression and burnout predict changes in eating competence, cognitive restraint, emotional eating, uncontrolled eating, and physical activity, and vice versa, using RI-CLPM. Based on prior research, we hypothesised bidirectional associations between mental health and health behaviours.

## Methods

### Study design

This randomised controlled trial (RCT) aimed to improve participants' health behaviours and promote weight loss. Participants were randomly assigned to one of the three online intervention groups: eHealth, eHealth + Group, or eHealth + Individual. Here, ‘eHealth’ refers to the use of *Healthy Weight Coaching (HWC)* online programme (Kupila et al., 2022; Suojanen et al., 2020), with two intervention arms receiving additional support through either group (eHealth + Group) or individual sessions (eHealth + Individual). The intervention lasted 12 months (March 2021 to March 2022). To ensure sufficient statistical power, participants from all intervention arms were combined for the analyses, focusing on longitudinal changes over the 12-month intervention period rather than between-group differences. This approach was supported by prior analyses that did not indicate statistically or clinically meaningful differences between the treatment arms in attrition, or in depression, burnout, or health behaviour outcomes (Männistö and Pietiläinen, 2025a; Männistö and Muotka, 2025b).

The programme incorporated themes like diet, physical activity, sleep, and stress management, combined with elements from Acceptance and Commitment Therapy (ACT) such as values, committed action, and mindfulness. The goal was to develop weight management skills and improve psychological flexibility to support long-term weight loss.

Participants were instructed to complete one online session per week. Personalised feedback was provided every two weeks for the first three months and every three weeks thereafter by a designated coach who had advanced degrees in nutrition and training in psychology.

Participants were recruited in February and March 2021 from Helsinki city employees with a BMI of 30–40 kg/m^2^ via internal advertisements and recommendations from Occupational Health Care Helsinki. Participants underwent a preliminary telephone screening to assess study eligibility, including internet access and the ability to attend group or individual sessions; exclusion criteria comprised involvement in other weight loss programmes, recent pregnancy, significant weight fluctuations, serious health conditions, or use of weight loss medication.

Following a preliminary screening, candidates completed a phone-based health assessment with a medical doctor. After obtaining informed consent, participants underwent laboratory tests and body composition measurements before registering for the online programme. Participant assessments were conducted at baseline (0 mo), mid-intervention (6 mo) and post-intervention (12 mo) between March 2021 and March 2022. Questionnaires were delivered as a part of the online intervention at the specified time points regardless of the participants’ progress in the programme.

The study received ethical approval from the Helsinki Uusimaa Hospital District (HUS/922/2020; date: April 29, 2020) and was conducted in accordance with the Declaration of Helsinki guidelines.

#### Outcome measures

**Depressive symptoms** were assessed using the validated Finnish version (Nuevo et al., 2009) of the 21-item Beck Depression Inventory (BDI-21; Beck et al., 1996). BDI-21 is a widely-utilised tool with strong psychometric properties (Shafer, 2006), covering both cognitive (*e.g. “I do not feel sad.—I am so sad and unhappy that I can't stand it.; I make decisions about as well as I ever could.—I can't make decisions at all anymore.)* and physical (*e.g. I can sleep as well as usual—I wake up several hours earlier than I used to and cannot get back to sleep.*) symptoms of depression (Beck et al., 1961). Each item was rated on a scale from 0–3, with a total score ranging from 0–63. Item 18 was modified to enquire about decrease *or increase* in appetite, scored equally. Item 19 was adapted to enquire about weight loss *or gain*. Considering participants’ involvement in a weight management programme weight loss was considered intentional and scored as zero. Higher scores correspond to more severe depressive symptoms. Scores of 0-12 indicate minimal or no depression, 13–18 mild depression, 19–29 moderate depression, and 30 or higher indicate severe depression.

**Burnout symptoms** were evaluated using the Finnish version of the Bergen Burnout Inventory (BBI-15; Näätänen et al., 2003) that has demonstrated good psychometric qualities (Näätänen et al., 2003; Salmela-Aro et al., 2011). BBI-15 measures the three core dimensions of burnout, each measured by five items: *exhaustion* (e.g. “I am snowed under with work”, “I often sleep poorly because of the situation at work”), *cynicism* (e.g. “I feel dispirited at work and I think of leaving my job”, “I am frequently annoyed with customers or other employees”, and *diminished professional efficacy* (e.g. “I frequently question the value of my work”, “Honestly, I felt more appreciated at work before” (Cooper et al., 2001, ). Each item is scored from 1 to 6 (1 = completely disagree, 6 = completely agree), yielding a total score between 15 and 90 where higher scores reflect more severe burnout.

**Eating competence** was evaluated using the Finnish version of the ecSatter Inventory 2.0 (ecSI2.0; Satter, 2007), a questionnaire with a confirmed construct validity (Lohse et al., 2018). The questionnaire consists of 16 statements covering *eating attitudes* (e.g. “I am comfortable about eating enough”, “I feel it is okay to eat food that I like”), *contextual skills* (e.g. ”I tune in to food and pay attention to eating.”, “I make time to eat”), *food acceptance* (e.g. I eat a wide variety of foods, If the situation demands, ”I can “make do” by eating food I don't much care for”, and *food regulation* (“I trust myself to eat enough for me.”, “I eat as much as I am hungry for.”). Responses are scored on a Likert scale from 0 to 3 (3 = always; 2 = often; 1 = sometimes, 0 = rarely; 0 = never, with a total score range of 0-48; higher scores reflect greater competence, with a score of 32 or more indicating good eating competence.

**Cognitive restraint, uncontrolled eating, and emotional eating** were measured using the 18-item Finnish version of the Three-Factor Eating Questionnaire (TFEQ-R18), which has demonstrated robust structural validity, reliability, and psychometric properties (Malkki-Keinänen et al., 2022). Items are scored from 1 to 4 (4 = definitely true, 3 = mostly true, 2 = mostly false, 1 = definitely false), where some items are reversed, and raw subscale scores are computed as the average of subscale items multiplied by the number of items, yielding a converted score as a percentage of the maximum possible raw score (0-100), with higher percentages indicating greater levels of cognitive restraint, uncontrolled eating, or emotional eating (Karlsson et al., 2000). Cognitive restraint is measured with six statements (e.g. “I deliberately take small helpings as a means of controlling my weight.”, “I do not eat some foods because they make me fat.”, uncontrolled eating is measured with nine questions (e.g. “Sometimes when I start eating, I just can’t seem to stop.”, “I get so hungry that my stomach often seems like a bottomless pit.”, and emotional eating is measured with three statements (e.g. “When I feel anxious, I find myself eating.”, “When I feel blue, I often overeat.”).

**Physical activity** was assessed using the Baecke Habitual Physical Activity Questionnaire (BHPAQ; Baecke et al., 1982), which covers *work* (e.g. “At work, I walk…”, “At work I sit...”), *sports* (e.g. “Which sports do you play most frequently?—How many hours a week?—How many months a year?”, and leisure activity (e.g. “How many minutes per day do you walk and/or cycle to and from work, school and shopping?”, “During leisure time, I watch television…”. The 16-item questionnaire is, rated on a scale from 1 to 5, with some items scored reversely. For the scoring of sports questions, a formula: (Intensity_1_*Time_1_*Proportion_1_) + (lntensity_2_*Time_2_*Proportion_2_) is used. The total score is the sum of the three subscales, with a higher score indicating greater levels of physical activity.

#### Statistical analysis

**Figure 1.**
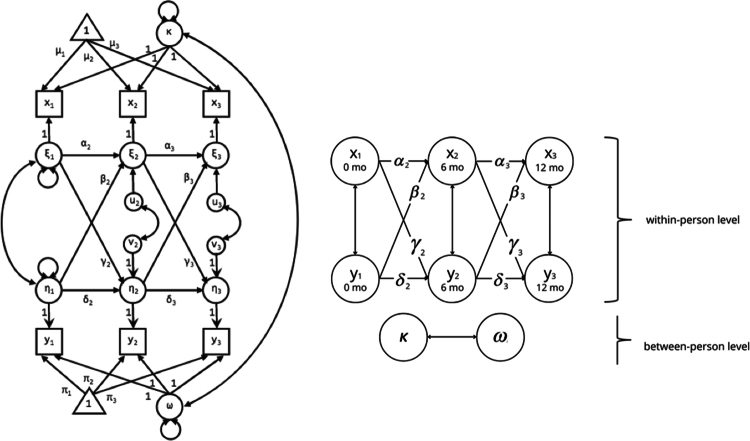
The full random intercept cross-lagged panel model (RI-CLPM) as presented by Hamaker et al. (2015) is presented on the left, with a simplified version, as presented in the result section, is on the right. In the full model x and y represent two variables measured at three different time points (_1,2,3_). The lines from the triangles to x and y—*μ* and *π*—indicate group means at each time point. The circles labeled *κ* and *ω* represent individuals’ trait-like deviations from these temporal group means, i.e. random intercepts, while the arrow between κ–ω represents the covariance between these between person-differences. On the within-person level, *ξ* and *η* represent individuals’ deviations from the group means at each time point, *α* and *δ* represent the carry-over effects, indicating how stabile is the individuals’ deviation from the group mean between the time points, and—crucially—cross-lagged parameters *γ* and *β* indicate “the degree by which deviations from an individuals’ expected score on y can be predicted from preceding deviations from one’s expected score on x, while controlling for the individuals’ deviation of the preceding expected score on y” (Hamaker et al., 2015), i.e. the extent in which the variable pair influences each other.

Random intercept cross-lagged panel model (RI-CLPM) was used to study the causal relationship between the mental health variables (depression, burnout) and health behaviour variables (eating competence, controlled restraint, emotional eating, uncontrolled eating and physical activity) in the longitudinal form (Hamaker et al., 2015) (Figure 1). RI-CLPM separates within-person and between-person variance, ensuring that observed cross-lagged relationships are not confounded by unmeasured, stable differences between individuals. The analysis model is especially valuable in the intervention setting, because, instead of reporting general associations, it shows how individual changes in one variable impact another.

To determine the optimal model structure for each pair of variables, we first ran the full RI-CLPM for all variable pairs. If the initial results indicated a need for refinement, we then conducted additional iterations of the model. Following the guideline set by Hamaker et al., we constrained the autoregressive and cross-lagged parameters if the results indicated that the effects of the variables on one another were stable. This approach increased the degrees of freedom and reduced the number of parameters, resulting in a simpler and more accurate parameter estimates in a small sample size. Model suitability was evaluated following Hu & Bentler, (1999) guidelines, with the fit information for final models detailed in Supplementary Table 1. The suitability of the restricted models was further assessed using the MLR sequential test (Satorra–Bentler formula; Satorra & Bentler, 2010), with the results shown in Supplementary Table 2. All reported models were suitable and had good model fit. Although the restricted model for the variable pair of burnout and physical activity demonstrated acceptable fit, the unrestricted model revealed cross-lagged effects in opposite directions. These paths were therefore left freely estimated, and the unrestricted model was used for reporting.

As a sensitivity analysis, intervention group was included as a covariate in the RI-CLPM models; however, this did not meaningfully change the observed associations. Due to the relatively small sample size and the complexity of the RI-CLPM framework, demographic variables, baseline BMI, and intervention engagement were not included as covariates in the RI-CLPM or CLPM analyses to avoid excessive model complexity and unstable parameter estimation.

Noteworthy, for the variable pair of burnout and emotional eating, the random intercept variance of emotional eating was negative and non-significant, strongly indicating a lack of between-person variance. Hence, to avoid over-estimation, we report the model from which the random intercept of emotional eating was removed. For the variable pair of depression and emotional eating, the RI-CLPM model could not be converged. Therefore, for this one variable pair, we present results using the CLPM model without random intercepts, although we were unable to test the suitability of this model compared to the RI-CLPM model.

Full information maximum likelihood estimation (FIML) with robust standard errors (MLR) was applied. Missing data were assumed to be missing at random (MAR). All the available data were included in the analyses. The sample size was determined through a priori power analysis conducted by a statistician (JM) and was based on anticipated weight change (the primary variable of the broader research framework).

Baseline correlations were examined using Spearman’s rank correlation coefficients, and differences between participants with complete versus missing follow-up data were analysed using analysis of variance (ANOVA). Internal consistency of the study measures was evaluated using Cronbach’s alpha coefficients at baseline, 6 months, and 12 months when item-level data were available. The coefficients are reported in Supplementary Table 4. Cronbach’s alpha could not be calculated for the TFEQ-R18 subscales because item-level responses were not available; only pre-calculated subscale scores were accessible for the present analyses.

Mplus version 8.11 was used for the statistical analysis. The figures were created using Canva, with the original model's structure adapted and simplified following the approach by Masselink et al. (2018).

## Results

### Participant characteristics

Of the 111 participants randomised into the study groups, 110 completed the baseline questionnaires and were included in the analyses. Participant characteristics and questionnaire scores at baseline, 6 months, and 12 months are presented in Table 1. At baseline, the mean age of the participants was 50.8 years and the mean BMI was 34.2 kg/m^2^. Most participants were women (82.7%), and the majority (87%) worked in white-collar occupations (manager, specialised expert, expert, or office and customer service worker). All participants were employed and identified as Finnish and white.

**Table 1.** Characteristics of participants (mean ± SD) who completed questionnaires at baseline, 6 months, and 12 months.

	Baseline (*N* = 110)	6 months (*N* = 90)	12 months (*N* = 58)
% male	17.3	12.2	13.8
Age	50.8 (8.89)	50.9 (8.70)	51.9 (8.39)
BMI (kg/m^2^)	34.21 (2.90)	33.78 (3.12)	33.04 (3.12)
Depression (BDI-21)	10.13 (7.27)	9.24 (6.73)	6.79 (6.37)
Burnout (BBI-18)	39.68 (14.12)	43.86 (15.04)	39.53 (13.09)
Eating competence (esCI)	23.20 (7.90)	25.00 (8.43)	28.23 (7.94)
Controlled restraint (TFEQ-CR)	38.44 (16.80)	50.69 (17.90)	46.41 (16.84)
Emotional eating (TFEQ-EE)	50.88 (22.84)	47.38 (20.68)	46.88 (19.01)
Uncontrolled eating (TFEQ-UE)	44.75 (19.05)	33.76 (19.23)	29.70 (18.51)
Physical activity (BHPAQ)	10.86 (1.60)	11.10 (1.33)	11.47 (1.37)

Most missing follow-up data were due to unanswered questionnaires (13% at 6 months and 34% at 12 months), whereas withdrawal from the study itself was rare (5% and 13%, respectively). Separate analyses for BBI and BDI retention showed that participants with missing questionnaire data did not differ from those with complete data in terms of age, baseline BMI, sex, treatment arm allocation, or baseline values of other questionnaire measures. The internal consistency of the available measures ranged from acceptable to excellent across assessment points. Cronbach’s alpha values were .862–.867 for depressive symptoms, .904–.927 for burnout symptoms, .819–.875 for eating competence, and .741–.780 for physical activity.

At baseline, depressive symptoms were moderately correlated with burnout (r = 0.56, *P* < .001) and weakly correlated with emotional eating (r = 0.23, *P* = .029) (Table 2). Burnout was also weakly associated with uncontrolled eating (r = 0.19, *P* = .047).

**Table 2.** Spearman correlation matrix of modelled variables at baseline, with numbers in the top row corresponding to the variables listed in the first column.

	Depr.	Burnout	EC	CR	EE	UE	PA
Depression		.56***	−.18	−.03	.23*	.18	.07
Burnout	.56***		−.15	.03	.19	.19*	−.05
Eating competence (EC)	−.18	−.15		−.09	.14	−.05	.01
Controlled restraint (CR)	−.03	.03	−.09		−.01	−.37***	.23*
Emotional eating (EE)	.233*	.19	.14	−.01		.34^**^	.09
Uncontrolled eating (UE)	.18	.19*	−.05	−.37***	.34**		−.20*
Physical activity (PA)	.07	−.05	.01	.23*	.09	−.20*	

^*^ *P* < .05.

^**^ *P* = .001.

^***^ *P* < .001.

#### Depression and health behaviour

**Figure 2.**
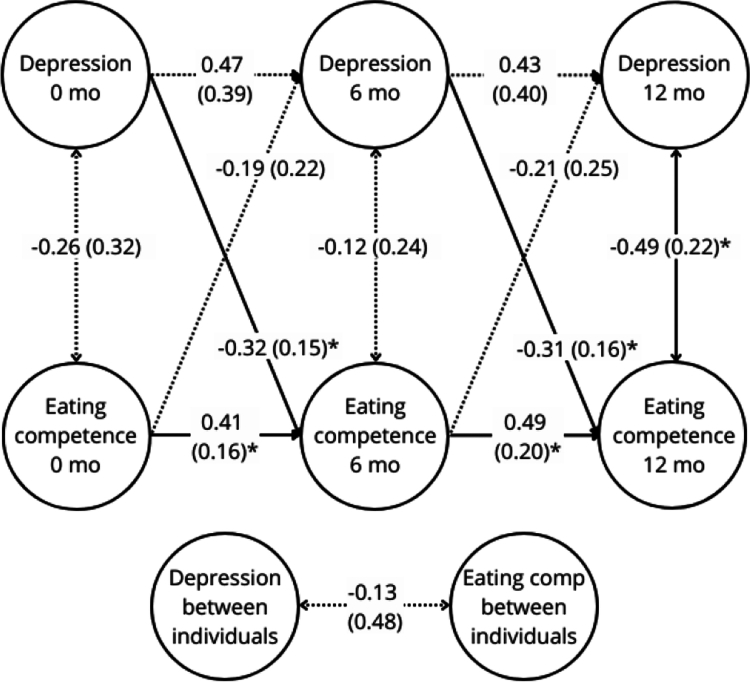
Simplified RI-CLPM model illustrating the longitudinal relationships between depression and eating competence. The model presents standardised coefficients and standard errors, with solid lines indicating statistically significant associations (*P < .05).

Higher depressive symptoms predicted subsequent changes in eating behaviour during the intervention. Specifically, the cross-lagged parameters from depression at baseline to eating competence at 6 months (γ2 = −0.32, *P* = .032) and from depression at 6 months to eating competence at 12 months (γ3 = −0.31, *P* = .046) were negative and statistically significant (Figure 2, Supplementary Table 3). In contrast, the parameters from eating competence to later depressive symptoms were not significant, indicating that depressive symptoms preceded changes in eating competence rather than the reverse.

Eating competence itself showed temporal stability across the intervention. Significant carry-over effects were observed from baseline to 6 months (δ2 = 0.41, *P* = .012) and from 6 months to 12 months (δ3 = 0.49, *P* = .011). The random intercept variances were 25.56 (*P* = .13) for depression and 22.63 (*P* = .24) for eating competence, and the covariance between the random intercepts was −0.13 (*P* = .79), indicating no stable between-person association between depression and eating competence across time.

**Figure 3.**
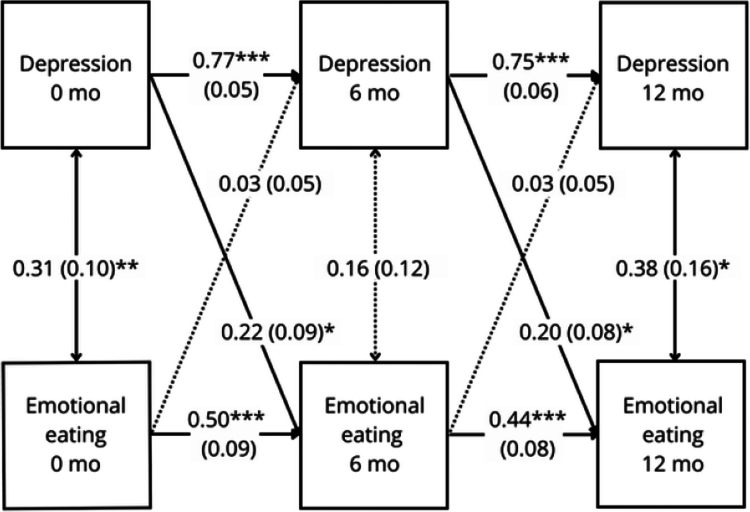
Simplified CLPM model showing the longitudinal relationships between depression and emotional eating. The model presents standardised coefficients and standard errors, with solid lines indicating statistically significant associations (***P < .001, **P < .01, *P < .05).

For the depression–emotional eating pair, results are based on a traditional CLPM model rather than RI-CLPM due to convergence issues. A similar directional pattern was nevertheless observed for emotional eating. Depressive symptoms predicted higher emotional eating at subsequent time points, with significant cross-lagged parameters from baseline depression to emotional eating at 6 months (γ2 = 0.22, *P* = .013) and from depression at 6 months to emotional eating at 12 months (γ3 = 0.20, *P* = .014) (Figure 3, Supplementary Table 3). The reverse paths from emotional eating to later depressive symptoms were not significant (β2 = 0.03, *P* = .55; β3 = −0.03, *P* = .55).

Both depressive symptoms and emotional eating showed stability across time. Depression demonstrated carry-over effects from baseline to 6 months (α2 = 0.77, *P* < .001) and from 6 months to 12 months (α3 = 0.75, *P* < .001). Emotional eating also showed stability between baseline and 6 months (δ2 = 0.50, *P* < .001) and between 6 months and 12 months (δ3 = 0.44, *P* = .025).

No significant longitudinal associations were observed between depressive symptoms and cognitive restraint or between depressive symptoms and physical activity (Supplementary Table 3). For uncontrolled eating, a carry-over effect was observed from baseline to 6 months (δ2 = 0.72, *P* = .019), but no cross-lagged associations with depression were detected.

Overall, these findings indicate that increases in depressive symptoms preceded decreases in eating competence and increases in emotional eating during the intervention period.

#### Burnout and health behaviour

**Figure 4.**
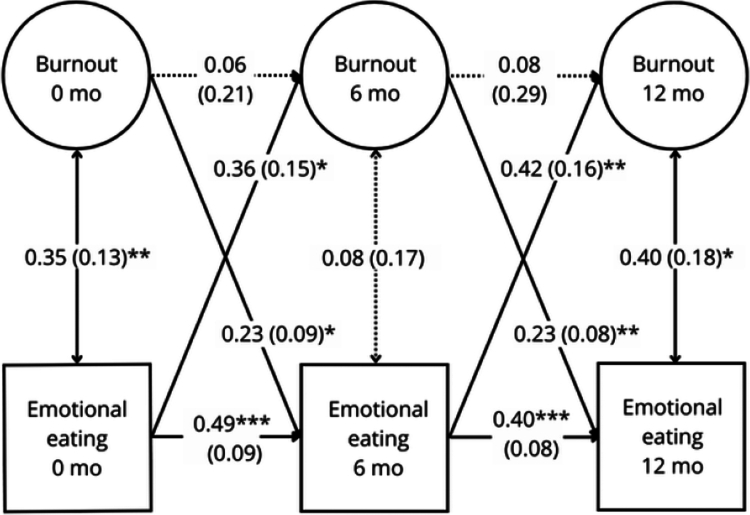
Simplified model illustrating the longitudinal relationships between burnout and emotional eating. The random intercept for the emotional eating variable has been removed to improve model precision. Circles denote latent variables, while squares represent observed variables. The model presents standardised coefficients and standard errors, with solid lines indicating statistically significant associations (***P < .001, **P < .01, *P < .05).

Burnout symptoms were longitudinally associated with emotional eating. The cross-lagged parameters from burnout at baseline to emotional eating at 6 months (γ2 = 0.23, *P* = .014) and from burnout at 6 months to emotional eating at 12 months (γ3 = 0.23, *P* = .007) were positive and statistically significant (Figure 4, Supplementary Table 3). In addition, the relationship was bidirectional: emotional eating also predicted later burnout symptoms, with significant paths from emotional eating to burnout (β2 = 0.36, *P* = .016; β3 = 0.42, *P* = .007).

Emotional eating showed temporal stability between baseline and 6 months (δ2 = 0.49, *P* < .001) and between 6 and 12 months (δ3 = 0.40, *P* < .001). The random intercept variance was 105.49 (*P* < .001) for burnout, whereas the random intercept for emotional eating was removed in the final model due to lack of between-person variance.

**Figure 5.**
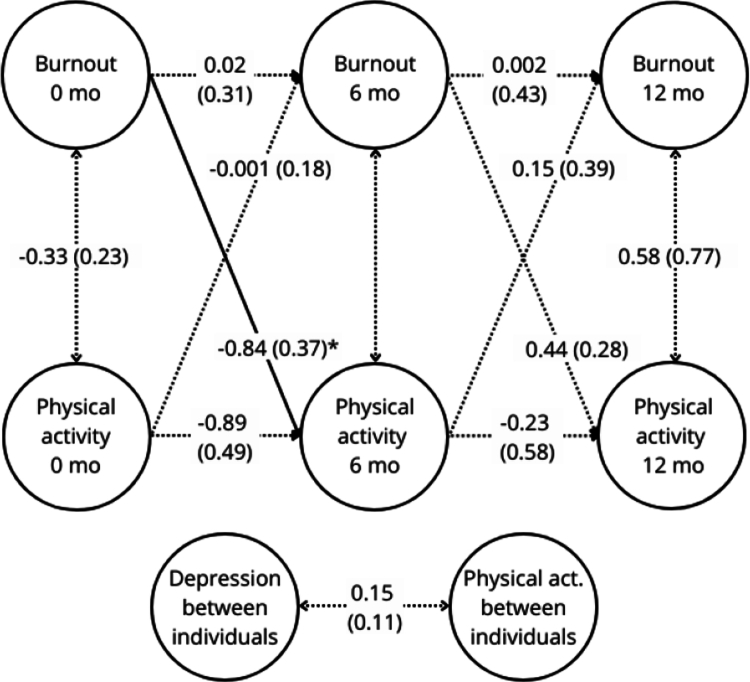
Simplified RI-CLPM model illustrating the longitudinal relationships between burnout and physical activity. The model retains standardised coefficients and standard errors from the full model, with solid lines indicating statistically significant associations (*P < .05).

Burnout was also associated with later physical activity. The cross-lagged parameter from burnout at baseline to physical activity at 6 months was negative and statistically significant (γ2 = −0.84, *P* = .023) (Figure 5, Supplementary Table 3). However, no significant cross-lagged effects were observed from burnout to physical activity between 6 and 12 months, nor from physical activity to burnout at any time point. No within-person carry-over effects were observed for this variable pair.

The random intercept variances were 124.72 (*P* < .001) for burnout and 1.86 (*P* < .001) for physical activity. The covariance between the random intercepts was 0.15 (*P* = .17), indicating no stable association between individuals’ overall levels of burnout and physical activity.

No significant longitudinal associations were observed between burnout and uncontrolled eating (Supplementary Table 3). Minor stability effects were detected for eating competence between baseline and 6 months (0.45, SE = 0.21, *P* = .030) and for cognitive restraint between 6 and 12 months (0.45, SE = 0.23, *P* = .048), but no cross-lagged effects between burnout and these behaviours were observed.

Overall, the results indicate a bidirectional relationship between burnout and emotional eating, with both variables predicting increases in the other over time. In addition, higher burnout symptoms at baseline predicted lower physical activity six months later.

## Discussion

This study examined whether changes in depressive and burnout symptoms precede changes in eating behaviour and physical activity during a 12-month behavioural obesity intervention among occupational health care clients. The findings suggest that psychological distress may undermine key health behaviours required for successful weight management. Specifically, increases in depressive symptoms preceded decreases in eating competence and increases in emotional eating, while increases in burnout symptoms preceded increases in emotional eating and decreases in physical activity. These findings highlight psychological distress as a potential barrier to sustained behavioural change among working-age adults with obesity.

Our results show that individuals’ higher depressive symptoms at previous time point predicted lower eating competence subsequently. Confirmed by a recent systematic review article by Eaton et al. (2024), there are no previous studies investigating the relationship between clinical depression and eating competence. However, intuitive and mindful eating, which can be considered equivalent to subcomponents *contextual skills* and *food regulation* of *eating competence*, have significantly been associated with lower levels of depression (Carrard et al., 2021; Lee et al., 2020; Winkens et al., 2018). Only one study, Hazzard et al. (2021), explored longitudinal associations. They found that higher levels of intuitive eating predicted lower probability of high depressive symptoms.

Furthermore, we found that higher depressive symptoms at previous time point predicted increased emotional eating subsequently. This aligns with extensive research showing that elevated depressive symptoms are associated with higher levels of emotional eating (Dakanalis et al., 2023; Goldschmidt et al., 2013; Konttinen et al., 2010; Konttinen et al., 2019; Paans et al., 2018; van Strien et al., 2016). While most previous research is cross-sectional, a large-scale, 18-year study by Vittengl (2018) provides evidence that depression and emotional eating have a longitudinal, bidirectional relationship in women. This is partially against our finding suggesting that depression is the dominating variable predicting emotional eating, and not vice versa. Our small sample size and inability to analyse sexes separately could have impacted the result. Contradicting results from a previous group level study (Paans et al., 2018), our data did not show depressive symptoms to be associated with uncontrolled eating or cognitive restraint.

Considering occupational burnout, we observed that reported increase in burnout symptoms predicted increased emotional eating at subsequent time points. The longitudinal association was bidirectional: elevated levels of emotional eating also predicted later burnout symptoms. The association aligns with previous research showing that baseline burnout is associated with higher levels of emotional eating (Kristanto et al., 2016; Nevanperä et al., 2012). It is often theoretically assumed that burnout predisposes to emotional eating but our results show that also heightened levels of emotional eating can precede burnout. Since food can provide short-term comfort in high-stress situations (Tomiyama et al., 2011), and individuals under the highest stress levels are more prone to emotional eating (Järvelä-Reijonen et al., 2016), emotional eating might serve as an early indicator of burnout risk, signalling stress levels that could contribute to its progression.

Moreover, we observed that individuals’ heightened burnout level at baseline was associated with decreased physical activity at 6 months. This result aligns with previous group-level results showing that burnout, especially the exhaustion dimension, is linked with infrequent exercise (Alexandrova-Karamanova et al., 2016). Previous studies show that physical activity plays a preventive role against burnout, helping reduce burnout risk over time (Naczenski et al., 2017; Shoman et al., 2021). However, our findings indicate a different pattern: we observed only an association in the reverse direction, with burnout predicting physical activity levels rather than activity having a clear protective effect against burnout symptoms. This suggests that, at least in our data, individual fluctuation in burnout symptoms influence physical activity engagement rather than the other way around.

Against our hypothesis, we did not observe longitudinal associations between depression and physical activity. This is surprising since extensive literature shows that higher levels of physical activity protect against depressive symptoms (Gianfredi et al., 2020; Pearce et al., 2022). Also, depression has been associated with decreased physical activity, especially among women and individuals over the age of 40 (Martinsen, 2008), who constitute most our study group. Furthermore, we did not discover longitudinal associations between depression or burnout with uncontrolled eating. The lack of significant associations misaligns with previous research showing that higher depressive symptoms and baseline burnout symptoms are linked with a greater tendency for uncontrolled eating (Nevanperä et al., 2012; Paans et al., 2018).

Several characteristics of the present study should be considered when interpreting the findings. First, the data were derived from a three-arm intervention study. Pooling participants across intervention arms assumes broadly similar underlying processes across groups, an assumption supported by the absence of systematic differences in key variables and by sensitivity analyses including intervention group as a covariate. Because the models focused on within-person associations over time, pooling is unlikely to have substantially biased the estimated relationships, although subtle group-specific effects cannot be entirely excluded.

Second, the observed longitudinal associations emerged within an active ACT-informed behavioural intervention rather than under naturalistic conditions. Consequently, the identified temporal relationships likely reflect psychological and behavioural processes occurring in the context of ongoing weight management efforts, coaching support, and repeated self-monitoring. Intervention components emphasising mindfulness, values-based behaviour change, stress management, and eating-related awareness may have influenced how depressive and burnout symptoms interacted with eating behaviour and physical activity over time. Moreover, participant motivation and engagement, together with the supportive coaching environment, may have modified the strength of these associations. Although RI-CLPM accounts for stable between-person differences through random intercepts, the present models did not account for individual differences in intervention-related change trajectories. Therefore, the findings should be interpreted within the context of behavioural obesity treatment rather than as fully generalisable naturalistic associations.

Several methodological limitations should also be acknowledged. The relatively small sample size and missing data may have reduced statistical power, increasing the likelihood that true associations remained undetected. Furthermore, the a priori power calculations for the broader trial were based on anticipated weight change rather than the longitudinal associations examined here. Consequently, the analyses may have been underpowered to detect smaller within-person effects, increasing the risk of false negative findings and reducing model stability. Conversely, the large number of tested associations increases the possibility of false positive findings. Although missing data were addressed using full information maximum likelihood estimation, robust standard errors, inclusion of all available observations, and sensitivity analyses, missingness may nevertheless have reduced the reliability of the results. Accordingly, the findings should be considered exploratory and require replication in larger samples.

The sample consisted primarily of employed, highly educated White women working in expert professions, which limits the generalisability of the findings. In addition, a convergence issue prevented the use of RI-CLPM for the depression–emotional eating association, necessitating the use of a traditional CLPM model. This result should therefore be interpreted more cautiously, as CLPM does not separate within-person variation from stable between-person differences and may overestimate longitudinal associations due to confounding by trait-like characteristics. Nevertheless, CLPM remains a widely used approach for examining directional longitudinal relationships (Hamaker et al., 2015), facilitating comparison with previous studies. Similarly, the need to retain freely estimated cross-lagged paths in the burnout–physical activity model may have reduced model stability, warranting cautious interpretation.

The study further relied on self-reported measures, which may have introduced reporting bias. In addition, the depression measure did not distinguish between melancholic and atypical depression. Because atypical depression is associated with increased appetite (Łojko & Rybakowski, 2017), female sex, and higher BMI (Koning et al., 2022), the observed association between depression and emotional eating may primarily reflect characteristics of this subtype, which accounts for approximately one-third of depression cases (Łojko & Rybakowski, 2017). Future studies should examine whether the observed associations differ between depressive subtypes. Likewise, burnout was analysed as a global construct, preventing assessment of whether specific dimensions were responsible for the observed effects. Although burnout is multidimensional (Schaufeli et al., 2009), exhaustion is generally regarded as its core component (Brenninkmeijer & Van Yperen, 2003) and may be particularly relevant for reductions in physical activity. However, associations between burnout and eating behaviour may also involve cynicism and reduced professional efficacy.

The study was conducted during the COVID-19 pandemic (2021–2022), a period that may have influenced eating behaviour, physical activity, psychological well-being, and work-related strain. Pandemic-related disruptions, including changes in work arrangements, social isolation, and reduced opportunities for physical activity, may have contributed to increased stress, burnout symptoms, emotional eating, and lower physical activity (Almandoz et al., 2022). Previous research suggests that individuals with higher BMI were particularly vulnerable to adverse lifestyle changes during the pandemic (Robinson et al., 2021), whereas emotional eating remained relatively stable at the population level (Coulthard et al., 2021). Furthermore, individuals with greater depressive symptoms were more likely to change their eating behaviour during the pandemic (Herle et al., 2021). Although pandemic-related experiences were not directly measured, they may have influenced the observed longitudinal associations.

Despite these limitations, the study has several strengths. To our knowledge, this is the first study to investigate longitudinal within-person associations between depression and burnout symptoms and eating behaviour and physical activity. Strengths include the use of validated measures, repeated assessments, and advanced longitudinal modelling techniques that enabled the separation of within-person and between-person processes. In addition, model selection was tailored to each variable pair, and the adequacy of the final models was supported by satisfactory model fit indices and MLR sequential testing.

In conclusion, the present study suggests that fluctuations in psychological distress may act as an antecedent of changes in key health behaviours among adults with obesity participating in a behavioural weight management intervention. Increases in depressive symptoms preceded decreases in eating competence and increases in emotional eating, whereas increases in burnout symptoms preceded increases in emotional eating and reductions in physical activity. Notably, emotional eating also predicted subsequent burnout symptoms, indicating a potentially self-reinforcing cycle between work-related distress and maladaptive eating behaviour. Together, these findings suggest that psychological distress may impair behaviours that are central to successful weight management and long-term health improvement. Assessment and treatment of depressive and burnout symptoms may therefore be important components of obesity interventions, particularly among working-age adults. Future studies with larger samples are needed to confirm these findings and to clarify the mechanisms linking psychological well-being with eating behaviour and physical activity over time.

## Supplementary Material

Supplementary Material Supplementary_tables.docx

## Data Availability

The datasets generated or analysed during this study are available from the corresponding author on reasonable request.

## The use of generative Artificial Intelligence (AI)

ChatGPT (OpenAI, GPT-5-mini) was used for language refinement and editorial support, and Scopus AI as a supplementary resource to aid in identifying relevant literature. These tools supported literature searching and clarity of expression, with all outputs carefully reviewed and revised by the author to ensure accuracy and integrity. Importantly, AI was not used to perform, analyse, interpret, or derive insights from the research data. All substantive intellectual contributions and scholarly judgments remain the authors’ own.
