# Supplementary material for: Longitudinal associations of depressive and burnout symptoms with eating behavior and physical activity in occupational health clients with obesity
Source: Health Psychol Behav Med. 2026 Aug 5;14(1):2700076. doi: 10.1080/21642850.2026.2700076 (PMC13446057; doi:10.1080/21642850.2026.2700076)
Supplement: Supplementary file 1 [file RHPB_A_2700076_SM7318.docx]

Supplementary Table 1. The model fit information for final models presented in the results, with the RI-CLPM analysis conducted with Mplus version 8.11.

|  | Chi-square value | Df | P-value | RMSEA | TLI | CFI |
| --- | --- | --- | --- | --- | --- | --- |
| **Depression –** |  |  |  |  |  |  |
| **Eating competence** | **3.18** | **5** | **0.67** | **0.00** | **1.00** | **1.00** |
| **Emotional eatingª** | **12.61** | **8** | **0.13** | **0.07** | **0.97** | **0.94** |
| Uncontrolled eating | 3.16 | 5 | 0.62 | 0.00 | 1.00 | 1.00 |
| Cognitive restraint | 4.05 | 5 | 0.54 | 0.00 | 1.00 | 1.00 |
| Physical activity | 5.67 | 5 | 0.34 | 0.04 | 0.99 | 1.00 |
| **Burnout –** |  |  |  |  |  |  |
| Eating competence | 5.50 | 5 | 0.36 | 0.03 | 0.99 | 1.00 |
| **Emotional eating** | **9.03** | **7** | **0.25** | **0.05** | **0.98** | **0.96** |
| Uncontrolled eating | 7.03 | 5 | 0.22 | 0.06 | 0.96 | 0.99 |
| Cognitive restraint | 4.18 | 5 | 0.52 | 0.00 | 1.00 | 1.00 |
| **Physical activity** | **2.48** | **3** | **0.48** | **0.00** | **1.00** | **1.00** |

ª CLPM model.

Supplementary Table 2. MLR sequential test results for restricted models presented in the article, with non-significant P value indicating suitability of the restricted model.

|  | DF0*C0 | DF1*C1 | DF0-DF1 | cd | C0*CHI_H0 | C1*CHI_H1 | Difference | Sequential test | P value |
| --- | --- | --- | --- | --- | --- | --- | --- | --- | --- |
| Depression – Eating competence | 4.56 | 0.63 | 4 | 0.98 | 2.90 | 0.88 | 2.03 | 2.06 | 0.72 |
| Depression – Emotional eatingª | 8.61 | 4.55 | 4 | 1.01 | 13.57 | 7.20 | 6.37 | 6.28 | 0.18 |
| Depression – Uncontrolled eating | 5.37 | 0.64 | 4 | 1.18 | 3.78 | 0.82 | 2.96 | 2.50 | 0.64 |
| Depression – Physical activity | 4.35 | 0.33 | 2 | 2.01 | 4.93 | 3.50 | 1.43 | 0.71 | 0.70 |
| Burnout – Eating competence | 4.75 | 0.28 | 4 | 1.12 | 5.23 | 4.23 | 1.00 | 0.90 | 0.93 |
| Burnout – Emotional eating | 6.70 | 2.76 | 4 | 0.98 | 8.64 | 2.69 | 5.95 | 6.05 | 0.20 |
| Burnout – Uncontrolled eating | 5.65 | 0.39 | 4 | 1.32 | 7.94 | 4.92 | 3.03 | 2.30 | 0.68 |
| Burnout – Cognitive restraint | 4.45 | 0.75 | 4 | 0.93 | 3.72 | 0.12 | 3.60 | 3.89 | 0.42 |

ª CLPM model

Formula: (DF0*C0-DF1*C1)/(DF0-DF1) = cd; (C0*CHI_H0-C1*CHI_H1) = difference; difference/cd = sequential test, where

DF0 = degrees of freedom in the restricted model

C0 = ‘scaling correction’ value in the restricted model

DF1 = degrees of freedom in the unrestricted model

C1 = ‘scaling correction’ value in the unrestricted model

CHI_H0 = chi^2^ value in the restricted model

CHI_H1 = chi^2^ value in the unrestricted model

Supplementary Table 3. Comprehensive standardized model results of the random intercept cross-lagged panel model (RI-CLPM) conducted using Mplus version 8.11, with statistically significant cross-lagged relationships highlighted in bold and visualized in the main article.

|  | Cross-lagged parameters | | | | Carry-over effects | | | | Between-person variance | | |
| --- | --- | --- | --- | --- | --- | --- | --- | --- | --- | --- | --- |
|  | *γ_2_* | *γ_3_* | *β_2_* | *β_3_* | *α_2_* | *α_3_* | *δ_2_* | *δ_3_* | *Κ^∞^* | *ω^∞^* | *κ–ω* |
| *Depression –* |  |  |  |  |  |  |  |  |  |  |  |
| **Eating competence** | **-0.32^*^** (0.15) | **-0.31^*^**  (0.16) | **-0.19**  (0.22) | **-0.21**  (0.25) | **0.47**  (0.39) | **0.43**  (0.40) | **0.41^*^** (0.16) | **0.49^*^**  (0.20) | **25.56** (16.81) | **22.63** (19.35) | **-0.13** (0.48) |
| **Emotional eatingª** | **0.22^*^** (0.09) | **0.20^*^**  (0.08) | **0.03**  (0.05) | **0.03**  (0.05) | **0.77^***^** (0.05) | **0.75^***^** (0.06) | **0.50^***^**  (0.09) | **0.44^***^** (0.08) |  |  |  |
| Uncontrolled eating | 0.18  (0.20) | 0.12  (0.13) | 0.06  (0.36) | 0.05  (0.31) | 0.12  (0.52) | 0.08  (0.38) | 0.52  (0.49) | 0.49  (0.48) | 35.18^***^ (8.02) | 176.13 (213.96) | 0.20  (0.26) |
| Cognitive restraint | -0.26 (0.26) | -0.29 (0.30) | -0.34 (0.21) | -0.38 (0.22) | 0.29  (0.36) | 0.25  (0.33) | 0.29  (0.22) | 0.42  (0.35) | 28.45^*^ (12.15) | 117.92 (64.47) | 0.25  (0.47) |
| Physical activity | -0.61 (0.88) | -0.19 (0.27) | 0.05  (0.30) | 0.02  (0.09) | 0.14  (0.55) | 0.09  (0.43) | -0.63 (1.12) | -0.09 (0.06) | 35.45^***^ (9.48) | 1.83^***^  (0.33) | 0.20  (0.15) |
| *Burnout –* |  |  |  |  |  |  |  |  |  |  |  |
| Eating competence | 0.09  (0.16) | 0.11  (0.19) | 0.10  (0.21) | 0.15  (0.28) | 0.02  (0.18) | 0.03  (0.29) | 0.45^*^  (0.21) | 0.53  (0.27) | 123.34^***^ (23.43) | 27.67 (15.58) | -0.36 (0.26) |
| **Emotional eating^b^** | **0.23^*^ (0.09)** | **0.23^**^ (0.08)** | **0.49^***^ (0.09)** | **0.42^**^ (0.16)** | **0.06**  **(0.21)** | **0.08**  **(0.29)** | **0.49**  **(0.09)** | **0.40**  **(0.13)** | 112.15^**^ (38.96) | -562.36 (1502.47) | -31.42 (196.73) |
| Uncontrolled eating | 0.22  (0.17) | 0.22  (0.14) | 0.20  (0.16) | 0.28  (0.26) | 0.07  (0.19) | 0.10  (0.28) | 0.38  (0.34) | 0.34  (0.29) | 111.47^***^ (23.93) | 235.56^*^ (91.96) | 0.35  (0.19) |
| Cognitive restraint | 0.04  (0.11) | 0.06  (0.16) | 0.02  (0.17) | 0.03  (0.29) | -0.004 (0.16) | -0.006 (0.26) | 0.36^*^  (0.17) | 0.55^*^  (0.26) | 126.49^***^ (20.50) | 127.77^*^ (58.49) | -0.04 (0.18) |
| **Physical activity** | **-0.84^*^ (0.37)** | **0.44**  **(0.28)** | **-0.001 (0.18)** | **0.15**  **(0.39)** | **0.02**  **(0.31)** | **0.002 (0.43)** | **-0.89 (0.49)** | **-0.23 (0.58)** | **124.72**^***^ **(26.18)** | **1.86**^***^  **(0.27)** | **0.15**  **(0.11)** |

*^∞^* from unstandardized model results

*ª using CLPM, instead of RI-CLPM*

*^b^ using a model in which the RI of emotional eating was removed; the RI variances from the original RI-CLPM model are displayed in between-person variances section*

*^*^P<0.05*

*^**^P<0.01*

*^***^P<0.001*

*γ_2_ = depression/burnout 0mo to health behavior 6mo*

*γ_3_ = depression/burnout 6mo to health behavior 12mo*

*β_2_ = health behavior 0mo to depression/burnout 6mo*

*β_3_ = health behavior 6mo to depression/burnout 12mo*

*α_2_ = depression/burnout 0mo to 6mo*

*α_3_ = depression/burnout 6mo to 12mo*

*δ_2_ = health behavior 0mo to 6mo*

*δ_3_ = health behavior 6mo to 12mo*

*κ = random-intercept variance of depression/burnout*

*ω = random-intercept variance of health behavior*

*κ–ω = covariance of the random-intercept variance*

Supplementary Table 4. Cronbach’s alpha coefficients (α) for the internal consistency of study measures at baseline, 6 months, and 12 months.

| Variable | Number of items | Baseline (0mo) α | Mid-intervention (6 mo) α | Post-intervention (12 mo) α |
| --- | --- | --- | --- | --- |
| BDI-21, depressive symptoms | 21 | .862 | .865 | .867 |
| BBI-15, burnout symptoms | 15 | .918 | .927 | .904 |
| ecSI2.0, eating competence | 16 | .819 | .875 | .856 |
| BHPAQ, physical activity | 16 | .757 | .780 | .741 |
| TFEQ-R18, cognitive restraint, uncontrolled eating,  emotional eating | 18 | N/A | N/A | N/A |

*N/A = not available; Cronbach’s alpha could not be calculated because item-level data were not available.*
